# Exploring the experience of young people receiving treatment for an eating disorder: family therapy for anorexia nervosa and multi-family therapy in an inpatient setting

**DOI:** 10.1186/s40337-022-00609-7

**Published:** 2022-07-12

**Authors:** Emily Coopey, George Johnson

**Affiliations:** 1 Centre for Applied Psychology, School of Psychology, University of Birmingham, Birmingham, UK; 2 Avon and Wiltshire Mental Health Partnership NHS Trust, Bristol, UK

**Keywords:** Adolescent, Anorexia nervosa, Qualitative research, Family therapy for anorexia nervosa, Multi family therapy

## Abstract

**Background:**

Research indicates that family therapy for anorexia nervosa (FT-AN) and multi-family therapy (MFT) are effective treatments for adolescents experiencing anorexia nervosa (AN). However, less is known about young people’s experiences of these two treatments, as there is limited qualitative research, and to date no qualitative research within an inpatient setting. It is argued that the lack of such insight limits the development of services for young people experiencing AN.

**Method:**

Five young people were recruited to the study from a specialist inpatient unit who were receiving treatment on the AN pathway which included both FT-AN and MFT. Semi structured interviews were undertaken and analysed using Interpretative Phenomenological Analysis.

**Results:**

Four superordinate themes and ten subthemes were developed from the data. The four superordinate themes were: ‘Process of Understanding’, ‘Reviving Connection’, ‘Emerging from the Eating Disorder and ‘Development of I’.

**Conclusions:**

There appeared to be two overarching concepts: the role of the individual and the role of others, that helpfully framed the results. The superordinate themes: ‘Emerging from the Eating Disorder’ and ‘Development of I’ focused on the development of the individual. Conversely, the superordinate themes: ‘The Process of Understanding’ and ‘Reviving Connection’ were centred on the relationships existing within the family system. The results could help inform future service developments regarding inpatient provision and service design.

**Plain English Summary:**

The most widely used and recognised treatment for anorexia nervosa in young people is family therapy for anorexia nervosa (FT-AN). An alternative treatment is multi-family therapy (MFT). Both treatments are deemed to be effective and usually happen in the community. However, some hospitals provide these treatments while the young people are in-patients. There is no research exploring young people’s experiences of these two treatments while in an in-patient unit.

Young people who had received both FT-AN and MFT in an inpatient setting were asked to share their experiences of these two treatments. Their stories were analysed by a researcher. The analysis identified four themes: ‘Process of Understanding’, ‘Reviving Connection’, ‘Emerging from the Eating Disorder and ‘Development of I’.

The results highlighted that the young people appeared to place more value on the role of others and perhaps others changing enabled them to change. The research highlighted the benefit in others’ understanding and therefore how improving societal understanding more broadly would be helpful. The young people reflected that both they and their parents benefited from FT-AN and MFT in an in-patient setting and it is proposed that this could help inform future service developments regarding inpatient provision.

## Background

### Anorexia nervosa

Anorexia nervosa (AN) is a serious condition associated with severe physical [1] and psychological complications [2], with one fifth of deaths associated with AN due to completed suicides [3]. AN is believed to have the highest mortality rate of all mental health conditions [4] and a higher mortality rate than physical health conditions such as asthma or diabetes [5].

### Anorexia nervosa in young people

AN is predominately associated with adolescence, and prevalence studies have shown the median age of onset for AN to be 12.3 years [6], with incidence rates being highest amongst the 15–19 age range [5]. AN is reported as the third most chronic illness amongst adolescents [5], this chronicity and severity highlights the importance of both effective and timely treatment as lower age and reduced duration of illness are factors associated with better treatment outcomes [7]. The ‘Access and Waiting Time Standard for Children and Young People with an Eating Disorder’ document states that young people should have access to National Institute for Health and Care Excellence (NICE) compliant treatment in a timely manner [8]. The document provides guidance on standards for establishing services and the delivery of treatment.

### Treatment for anorexia nervosa in young people

Family Therapy for Anorexia Nervosa^1^ (FT-AN) is the first line intervention for adolescents experiencing AN, as defined by NICE guidance [9]. FT-AN is a treatment that empowers parents to ‘re-feed’ their child, it is most commonly an outpatient treatment, delivered weekly to a single family facilitated by a single therapist. It consists of three phases which ultimately focuses on the young person taking back control of their eating. In addition to FT-AN, multi-family therapy (MFT) [10], was developed as a group treatment approach with different families coming together for treatment. Up to eight families may form group therapy sessions, consisting of a range of activities and tasks, usually with two therapists.

Research reviewing the existing literature highlights the efficacy of FT-AN and MFT [7]; FT-AN was deemed to be significantly better than the comparison treatment at six and twelve month follow ups [11] and MFT also resulted in significantly better treatment outcomes [12]. However, there remains a limited number of randomised control trials (RCTs) exploring the effectiveness of both treatments; a meta-analysis exploring adolescent treatment only included seven RCTs including FT-AN [13] and the most recent systematic review on MFT contained 27 studies [14], of which only two were RCTs [12, 15] and two were conducted in an inpatient setting [15, 16]. Research is supportive of the adaption of FT-AN [17–19] and MFT [15, 16] in more intensive treatment programs. To the authors knowledge there have been no studies focused on these combined treatments in inpatient settings and the evidence to support the effectiveness of inpatient treatment is variable and largely inconclusive [20].

We also note that there has been little qualitative research looking at adolescents’ experience of FT-AN. Some research has looked at adolescent experience of FT-AN in outpatient community settings [21–24], which highlighted the importance of family involvement. An even smaller body of qualitative research has been undertaken on young people’s experiences of MFT [25–27], highlighting treatment acceptability and the benefits of the group process. There are clear benefits to understanding how young people experience treatment [20], it is useful both to inform service development [28], and provide insight into treatment acceptability and complexity, [29]. For example, young people appeared to value FT-AN, finding key aspects of the manulised treatment most helpful, even if it was challenging for the them [29].Furthermore, the ‘Access and Waiting Time Standard for Children and Young People with an Eating Disorder’ document highlights the importance of commissioners understanding the experiences of young people in order to improve access to services [8].

## Method

### Aims of the project

The principal objective of this research was to explore young people’s experience of FT-AN and MFT in an inpatient setting in order to better understand the sense they make of the treatments provided and, from their perspective, treatment acceptability.

### Ethics

Ethical approval was granted by the Health Research Authority.

### Participants

Purposive sampling was used to recruit five participants to the study from a National Health Service inpatient unit for eating disorders (ED). The inpatient unit provided multiple treatment pathways, one being the AN treatment pathway which included having FT-AN and MFT as part of their inpatient treatment. Participants were recruited from the AN pathway; whilst on this individuals would receive manualised FT-AN and five days of MFT. Four of those days were held on consecutive weeks, with the fifth being held nearer to discharge. The MFT was a closed group with a maximum of eight families, facilitated by two clinicians. Individuals were referred to the inpatient unit via their community team as they were judged to be unable to remain at home due to the severity of their presentation. The young people were admitted and lived in the hospital unit without their family until they were deemed to be well enough to be discharged back to their community team. Family members were allowed to visit regularly, attended for therapy sessions and join other aspects of the unit’s routine, such as mealtimes and recreational activities. The participants had either a diagnosis of AN or a diagnosis largely characterised by features of AN, such as Other Specified Feeding and Eating Disorders. Four participants were female, and one was male, ages ranged from 10–18 with a mean age of 14.6 years. Three of the participants were nearing the end of their inpatient stay and were deemed to have completed MFT and FT-AN by the clinical team and the other two had recently been discharged. Pseudonyms have been allocated to participants to maintain confidentiality (see Table 1). The nature of the study meant that no additional demographics were collected regarding the participants. Although illness specific demographics were not obtained it is the case that participants conditions were sufficiently severe, at the time of admission, as to require inpatient care, and at the time of discharge they were well enough to be cared for by community teams.

**Table 1:** Participant demographics

Participant number	Name**
1	Morty
2	Lilly
3	Meghan
4	Lucy
5	Molly

**Pseudonyms have been allocated to maintain confidentiality

### Data collection

Participants engaged in semi-structured interviews designed to explore themes relating to the young person’s experience of treatment and of change. The interview guide was cocreated with an expert by experience. The interviews varied in duration, ranging from 24 to 76 min with a mean duration of 37 min.

### Data analysis

Interpretative Phenomenological Analysis (IPA) is a qualitative research method that focuses on examining how people make sense of their lived experiences [30]. It was particularly suited to this study as it was concerned with the sense young people make of their experience of receiving therapy in an inpatient unit. As IPA is concerned with the individual experience a small sample of participants was deemed appropriate [30], furthermore recruitment was limited due to the Covid-19 pandemic. Due to the interpretative nature of IPA and the existence of a ‘double hermeneutic’ (the researcher making sense of the participants own sense making), it was important to engage in reflexive processes. Due to the researcher’s (EC) previous experience of working in EDs and role as a trainee clinical psychologist, it was important for the researcher to remain mindful of her experiences to ensure that she did not map her understanding of reality on to those of the participants and make assumptions about their experiences, bracketing off assumptions and understanding. Many processes were incorporated throughout the progression of IPA to enable reflexivity and to enable consideration of biases: supervision, IPA tutorial groups, keeping a reflexive diary and discussions with supervisors and colleagues.

## Results

Four superordinate themes arose from the analysis of the data: ‘Process of Understanding’, ‘Reviving Connection’, ‘Emerging from the Eating Disorder’ and ‘Development of I’. Each superordinate theme had a number of subthemes (Table 2) which will be presented in further detail with associated quotes to support the development of the theme.

**Table 2:** Overview of themes

Superordinate Theme	Subthemes	Participants contributing
Process of Understanding	Understanding you are not alone	All participants
	Others understanding	All participants
	I am understood	All participants except Meghan
Reviving Connection	Altering the system	All participants
	Taking it forward	All participants
Emerging from the Eating Disorder	Understanding the immersion	All participants except Molly
	The Eating Disorder as damaging	Lily, Meghan and Molly
Development of I	Adapting to the new norm	All participants except Lilly
	The process of evolving	All participants
	Finding my voice	All participants except Lilly

## Process of understanding

The superordinate theme ‘Process of understanding’ related to the young people’s experience that once others came to understand more about the ED then the young person felt more understood.

### Understanding you are not alone

The theme ‘Understanding you are not alone’ arose from the sense that being with others, either professionals or peers, facilitated learning and change, as Lilly made sense of her experience of MFT: "I think it was nice to talk to other people, other parents, er, they knew they weren't alone and they gave them the tools to progress". Morty reflected on the impact of the groups on his parents’ understanding: "groups and stuff for like parents to understand and stuff … it helps like with my parents and stuff to understand and helps me to like try and deal with things a bit better”.

Throughout this theme there was an overarching sense that the young people attributed the benefit of the treatment to the impact it had on their parents’ understanding, as described by Molly: “it was almost Multi-Family Therapy was less for us, more for our parents, or at least that’s what we felt.” Despite the usefulness of connecting with others there was a sense that at times it could be unhelpful as Lucy explored: “…to hear people who had got re-admitted…”. Notwithstanding the apparent difficult nature of learning about relapse described by Lucy, it is questioned if this experience enabled a greater understanding of the ED for Lucy and her parents. It was felt that aiding understanding in others was seen as beneficial to the young people.

### Others understanding

When the young people talked about their experiences there was a sense that other people understanding more was central to their treatment experience, and this was enabled through the processes described in ‘Understanding you are not alone’. This notion related largely to parents having a better understanding of the ED: “I think that they’d say that [pause] their [pause] view of anorexia has changed probably … They see it as more a mental illness ……” as described by Lilly. There was a sense that the parents had gained a greater understanding of the illness itself but also the severity of the illness. The enhanced understanding appeared to sit within the context of supporting parents as described by Lucy: “it’s helped them” to understand the ED but Lucy still felt that her parents would never be able to fully understand her experience: “obviously you can never be 100%…”. This subtheme related to the young person valuing their parent having a greater awareness and understanding of the illness, which it felt enabled the young person to be better understood.

### I am understood

The subtheme ‘I am understood’ was created through the interpretation of the data which conveyed a sense that the young people were better understood as a result of their parents increased grasp of the ED. Lucy described her parents as having a greater understanding of the emotional impact: “Erm [pause] helped them understand the emotions associated with it [pause] and how it’s not one-sided ever. It’s always two sides”. Thus, sharing a sense that she is better understood and implying parental responses to her have been modified. The notion of modified parental responses was highlighted by Lilly who felt that her parents now knew how to support her: “Er, they know how to manage my meltdowns… and they know how to properly support me at mealtimes” and Molly described them as: “a lot more accepting”.

Morty described the idea that now his parents’ understanding had changed so had the way they made sense of his previous behaviour: “…I think she also thought like, about like I was choosing to be unwell and stuff but yeah.” Morty’s experience created a sense that his past behaviours were better understood in the context of the ED and raised the question as to whether there would be an improved interaction between Morty and his parents going forward due to this greater understanding of the young person. The increased understanding of the young person in the context of the ED appeared to highlight an improved interaction between the parents and young person.

## Reviving connection

The superordinate theme of ‘Reviving connection’ centred around the notion that there was a newness to the connection the young people and their families were experiencing. There was a process through which disconnection had occurred but altering the system through treatment enabled the system to act differently and thus revive the connection.

### Altering the system

The subtheme of ‘Altering the system’ centred on the idea that in order for the sense of disconnection to change then the system needed to be altered, with the alteration occurring through the interventions of FT-AN and MFT. The idea that someone external entering the system could facilitate change was offered by Molly: “…having someone a third party label it… makes it much easier to have conversations…I don’t sometimes have to say what I really think but they still understand what’s going on.” Lucy further supported the sense that an external voice entering the system was of benefit as it was believed to be positive: “… having a voice from someone else…you need someone else to kind of have an input.”. The role of an external entering the system seemed to centre on the benefit of an outside voice as well as the role of FT-AN and MFT as highlighted by Meghan: “… we did a role play … we were the parents [okay] and I think that sort of opened it up for a lot of us, sort of actually seeing sort of what it must be like in your parents’ perspective…”. The value in the role of treatment was situated in the context of enabling an alternative perspective. Meghan talks of understanding the situation from her parent’s perspective, and Molly talked of her family becoming: “a lot more aware of each other”. Supporting the notion that therapy has helped shift perspectives which has enabled the family system to act differently, as if the family scripts, which are the narratives the family hold are starting to become more fluid due to the input of others.

### Taking it forward

The shifts in family systems as a result of altered perspectives and increased understanding were acknowledged by the young people as they started to make sense of what this meant for their families in a longer term capacity, figuring out how to take the changes forward with them. The role of an external voice entering the system and shifting perspectives led to the familial systems acting differently. Young people talked of improvements in their relationships with significant others as Lilly describes: “getting closer again” to her sibling, which was supported by Meghan’s experience: “…not just like me and my family, my family’s got closer with each other as well…”. Molly explored the changes that had occurred for her family: “…being able to have more conversations, have like brother as an ally rather than someone to compete against.”, highlighting the revival of a sibling relationship and a sense that there is a version of normality emerging.

Although the young people reflected on these changes as positive, it was apparent that they were also challenging and required effort to embed in altered family scripts. Molly described: “…building on creating almost a new element of family is really difficult and still is…”. Molly highlighted the challenges faced by the family and the use of the word ‘building’ supports the notion that this process of change is hard work and takes effort and thought. There was a sense that despite the hard work implemented by the families there were times when the new way of doing things was ineffective. Lucy further highlighted the idea of ineffective communication; making sense of it as her family became stuck as they are: “…going round in circles…”.

There were further difficulties with embedding change within the family as highlighted by Morty who suggested that although talking was happening outside of therapy it was only: “sometimes” and: “not that much outside though”, highlighting the role of therapy in facilitating relational shifts. There was a sense that the young people wanted to take the changes forward, but it was a complex process. The impact of the individual on the shifts in the relational changes is important to consider and as this research explored the experiences of the young people their experiences of individual change will be explored further.

## Emerging from the eating disorder

The superordinate theme of ‘Emerging from the eating disorder’ resonated with the sense that at some point in the illness trajectory the young people experienced being consumed by the ED but as they progressed through treatment they came to gain a sense of perspective and to understand the ED as damaging.

### Understanding the immersion

The young people shared experiences of the ED, which were understood in the context of being immersed in the illness, as Lilly came to realise the need for inpatient treatment: “it got to a point where I wanted to get better and I knew I couldn’t do that at home”. Lilly describes feeling: “upset” by the experience of hospital but it feels as if she came to a realisation that she was so immersed in the ED that she had no other option; she could not recover at home implying the ED was too strong. The idea of struggling to progress was shared by Lucy who explained: “I wasn’t really making enough progress in the community. So it was, yeah, I was sent there”, implying inpatient treatment enabled her to emerge from the ED. 

There was a sense that the young people were fighting to return to a previous way of being, as Morty described: “they've helped with like, er, trying to get, er, back into like eating different foods.” The sense that Morty needed help to ‘try’ reinforces the gravity of the challenge the young people faced when trying to regain a sense of normality in the context of eating. Meghan used eating as a measurement of change: “ …I look back and realise how bad I used to actually …I’m eating now, but like a couple of months ago, I probably wouldn’t eat anything at all.”. The striking feature of Meghan’s account is the power in her realisation of how immersed she was; the young people needed facilitated recovery to enable them to reflect and realise the impact of the eating disorder on themselves. There is a sense that the ability to reflect on the past is key to their understanding and that the young people would not have understood the immersion at the time. The subtheme feels centred around the realisation of where one was in terms of the illness as opposed to evaluating the impact of the immersion.

### The eating disorder as damaging

There was a unanimous experience that the young people came to understand the illness as damaging; Lilly described it: “what it would look like it would be like spikey…” when trying to make sense of how she would externalise the ED. The word spikey is suggestive of causing harm to those on the exterior but perhaps also harmful to self as it ensures others are repelled and keep a distance. The notion of distance was supported by Lilly as she reflected an impact of the ED on her relationships: “We’re definitely not as close…, I really took it out on her.” Lilly highlighted the lasting impact it had on her sibling relationship whilst indicating she felt a sense of responsibility for these changes. Molly further contributed to this sense of damage caused to others and self as she described: “just the relief … you’re not forcing your parents into that situation anymore is incredible… it’s really difficult because you’re constantly lying”, this is suggestive of damage incurred due to the coercive nature of the ED but there is also a reinforcement of the responsibility or guilt that is experienced by the young people.

Molly further described the damage experienced by the ED in the context of damage to self: “I feel like I chose it because I did it to myself.”. Molly describes her experience as believing she made a choice in terms of her actions and it is felt that this is experienced as guilt for the impact of the ED; therefore providing an alternative stance on the ED as damaging as it appears to be damaging from an internal stand point for Molly. Interestingly, Molly questions if her beliefs about choice are: “an anorexic thought”, implying time might enable a shift away from her experience of responsibility. There was a sense that the young people experienced the ED as damaging due to the impact it had on others and the emotional impact on themselves because of the perceived sense of responsibility for the damage. However, there was a sense this could shift over time and this had occurred during their inpatient experience.

## Development of I

The superordinate theme of ‘Development of I’ was focused on experiences of individualised change for the young people. Throughout the theme there appeared to be a sense of questioning and almost hesitance in identifying and naming changes for them as individuals. It is important to consider the impact of individualised change on the system. Yet the value placed on change for the individual appeared less than that of the value placed on others changing and the systems changing.

### Adapting to the new norm

There was a sense that the young people experienced adjusting to a new sense of normal. Their experience of entering hospital was described as difficult, the young people felt they were entering the unknown, as described by Lucy: “It was hard…I just think being like taken away from your home is really difficult”. Molly reiterates the idea of the unknown: “…you’ve just suddenly moved into like a new place and you’re living with people [inaudible], so I was very scared when I first got here.”. Despite the sense that coming into hospital was challenging, Molly appears to be suggesting that this was her initial experience and it changed over time. 

The changes in an individual’s experience was attributed to the connection with others by Meghan: “…it was scary… then you make friends and … close relationships with people, [pause] it’s not as bad” whereas Morty seemed to make sense of it as: “it just feels a bit more like, just a bit more like you get used to it….” Thus there was an overarching awareness of the role of time that appeared to facilitate adaption, Lucy explained: “I think, as time went on, it became more and more valuable and different activities we did”, reinforcing the idea that time enabled the individual to develop to a point where they valued the input. The young people reflected on the challenging experience of entering an inpatient facility and how time enabled a shift in the way they felt, enabling an adaption.

### The process of evolving

The subtheme ‘The process of evolving’ related to the idea that there had been change for the young person as an individual and they experienced a process of change. Lucy recognised that over time her contribution to therapy evolved: “… throughout my stay I became more [pause] involved in the session”, it is questioned how the sense of involvement facilitated change for Lucy. Other young people noticed an emotional shift in themselves as Morty reflected: “…I’ve got more, er, calm with, er, sort of being in hospital, I guess…, the therapy like sort of changed how like I sort of felt as well…”. Although Morty recognised a shift in his internal state, he highlights the hesitance that surrounded the young people when discussing the evolution of themselves. It is questioned if this relates to a difficultly in expressing oneself or a reluctance to prioritise the role of I in this process. The idea of a change in one’s internal state is supported by Lilly: “My mental state is much different to the state it was in, feeling less upset, less tearful”, highlighting a positive evolution in her mental state.

Furthermore, the young people described a sense of evolving to be in a position where they better understood themselves as described by Meghan: “…it’s not just figuring out my problems, … I feel like [pause] I’m more confident, just as a social [yeah] person as well as before I came in here as well.” Meghan highlighted the benefits of her experience of treatment as not solely focused on the problem but a wider impact on the development of her as an individual, reflecting on how she evolved and how this differed from her former self. The young people reflected on an experience of evolving in their understanding of themselves, which led to a change in their internal states, and how they acted.

### Finding my voice

The final subtheme to be presented further explores the process of self-development and how the young people emerged from the process as an evolved version of their former selves. The idea that the young people had found a voice arose from the interpretation of the data as Meghan describes a change in how she approaches things: “… I wouldn’t dare to say anything before [yeah], I was very a keep to myself type of person, I can’t really do that here [laugh]”. There is a sense that Meghan was previously hesitant to use her voice and it feels as if the change in using her voice is associated with her sense of self-development. It is questioned if there was a sense of fear regarding using her voice and perhaps the power it held. There was a sense that practicing using their voices enabled them to find their voice as Lucy described: “… one of the things we did talk about in family therapy was how to deal with other people…now I can deal with it. So, I think that – talking about it definitely did help.”

There is a sense that by practicing using her voice it enabled it to become the norm and perhaps this impacted on the previously perceived repercussions of using their voices. Morty reinforces the notion that finding his voice, and subsequently using it, became the norm: “maybe it's just because, er, I’ve been with loads of people on the ward and talking to them, maybe I'm a bit more used to talking.” It is suggestive that through a process of finding their voices and talking, the young people had developed a sense of who they were and thus enabled them to share their lived experience of FT-AN and MFT in an inpatient setting.

## Discussion

Analysis of the data resulted in the development of four superordinate themes within two overarching concepts: *the role of the individual* and *the role of others*. The themes generated from this research highlighted that young people valued being understood and the revival of interrupted connection, they placed less emphasis on their experiences of change and the role of the individual within this experience.

### The role of the individual

The themes ‘Emerging from the eating disorder’ and ‘Development of I’ centre around the experience for the individual. The young people acknowledged the damaging nature of the ED in the theme ‘Emerging from the eating disorder’; which is consistent with Eaton [2] who described individuals as having a ‘pivotal moment of realisation’ regarding the damage caused by ED. However, the idea of a defining moment did not emerge from the transcripts; Eaton’s Meta-Ethnography is focused on the experiences of adult females and therefore it is possible that young people have a different experience. Existing research reported a deterioration in interpersonal relationships in the context of damage caused by the ED [2, 31–33], as opposed to damage to significant others and the impact on the individual in terms of responsibility for the damage caused to others as was evident in this research.

The young people explored the impact the ED had on their self-development, giving consideration to how they had evolved through the process during their reflections in the theme ‘Development of I’. Research supports the notion of an individual losing their sense of identity in an inpatient context [20, 34, 35], highlighting that inpatient settings can foster dependency [34]. However, the unique nature of the inpatient unit in terms of the use of FT-AN and MFT, combined with the encouraged parental involvement, may have prevented a sense of dependency developing.

The impact of individual change on the wider familial system could usefully be explored further as the young people participating in this research placed an emphasis on a shift in parental understanding, which is suggestive, that parental shift enabled individual change. It is believed systemic change, in the context of parental containment in FT-AN is needed in order to support individual change [24], reflecting the experience of the young people in this research. It is noteworthy that this still occurs for the individuals when undertaking FT informed treatments whilst removed from the family environment.

### The role of others

‘The process of understanding’, followed by ‘Reviving connection’ centred on others and focussed less on the individual. Within FT it is felt that an individual can be understood in the context of the familial system and this system is utilised to facilitate change [36], which is consistent with these themes.

A disconnect within the family was described by the young people in the theme ‘Reviving connection’, which is again supported by the literature. Research highlights the anxiety provoking nature of an ED and how this impacts on parents and consequently interconnection within the family [24]. Whitley and Eisler [37] reported that families adapt to centre around an illness, causing disruption to family life and magnifying existing patterns of family functioning. Therefore, the removal of the individual from the system in the context of an inpatient admission may disrupt this ‘centralisation of the illness’ and therefore provide a space to facilitate and enable change to occur.

The themes ‘The process of understanding’ and ‘Reviving connection’ were interconnected in the idea of learning and altering actions facilitated by the treatments. A Meta-Synthesis exploring young people’s experiences of FT-AN in community settings highlights the improvement in familial relationships via the process of therapy [29]. The notion of improved relationships was apparent in the experiences described by the young people in this research, highlighting similarities in the young people’s experiences of FT-AN in an inpatient context when compared with the existing literature. If young people detail a similar experience it is questioned if this supports the efficacy of the treatment outcomes as FT-AN and MFT are only researched in community settings.

### Clinical implications

A number of recommendations can be made in regard to service provision. The young people placed value in others learning and understanding about the ED which helped them to feel understood, and such training and education could form part of an intervention pathway, perhaps initially with further training and education to professionals. Individual ED services could provide training to primary care and third sector services to enhance recognition, understanding and signposting which could then be shared with parents and families.

The outcome of the research highlights treatment acceptability for young people in an inpatient setting. The young people appeared to value the treatments offered, and even when they did not value them for the benefit to themselves, they identified the value to parents. Furthermore, as the inpatient unit offers a novel way of delivering inpatient treatment the findings support the notion that young people benefit from this approach and value the support offered, recognising the value this could bring in terms of changing the family focus on illness.

At a wider level improving the societal understanding of EDs has potential clinical value as this may result in a more helpful response by parents when experiencing a child with an ED for the first time. In addition, ongoing support to parents was deemed valuable in this data set, which is supported by research evidence highlighting the acceptability of online support platforms [38] and anecdotal reports of the value of face-to-face support groups for parents.

### Limitations

There are limitations that need to be considered with this research project. The sample size was small, with a total of five participants, and whilst recruitment issues were unavoidable due to the service constraints and the Covid-19 pandemic, it does limit the findings and the potential for missing important experiences. However, qualitative research is typically associated with small sample size enabling rich, detailed accounts to be obtained through semi structured interviews providing insight into lived experience in a way that is unattainable in quantitative research. Furthermore, recruitment was carefully considered to ensure that a homogenous group of participants were recruited to enhance the understanding of this particular group. The value in a homogenous sample in qualitative research is that it enables an in-depth exploration of a particular group of people sharing a particular experience in a given context.

### Future research

Further research into the area of FT-AN and MFT in an inpatient setting would be of value to build on these findings, inform service provisions, as well as enhancing the understanding of young people’s experiences and acceptability of treatment modalities available to them.

## Conclusions

The research highlighted the process of evolving through treatment, for both the individual and their familial system. There was a sense that damage could be caused by the ED, yet treatment enabled repair. The research evidenced the importance of others learning and understanding EDs, which has implications for treatment services, as well as wider society.

## Data Availability

The datasets used and/or analysed during the current study are available from the corresponding author on reasonable request.

## Abbreviations

AN: Anorexia nervosa
ED: eating disorder
FT-AN: Family therapy for anorexia nervosa
IPA: Interpretative phenomenological analysis
MFT: Multi-family therapy
NICE: National Institute for Health and Care Excellence
RCTs: Randomised control trials

## Footnotes

1 Family Therapy for Anorexia Nervosa can also be referred to as Maudsley Family Therapy or Family Based Treatment.
